# Harnessing outer membrane vesicles derived from *Bordetella pertussis* to overcome key limitations of acellular pertussis vaccines

**DOI:** 10.3389/fimmu.2025.1655910

**Published:** 2025-09-02

**Authors:** Lucía Locati, Daniela Bottero, Francisco Carriquiriborde, Oriana López, Bernarda Pschunder, Eugenia Zurita, Pablo Martin Aispuro, Maria Emilia Gaillard, Daniela Hozbor

**Affiliations:** ^1^ Laboratorio VacSal, Instituto de Biotecnología y Biología Molecular (IBBM), Facultad de Ciencias Exactas, Universidad Nacional de La Plata, Centro Científico Tecnológico -Consejo Nacional de Investigaciones Científicas y Técnicas La Plata, La Plata, Argentina; ^2^ Instituto de Estudios Inmunológicos y Fisiopatológicos (IIFP), Centro Científico Tecnológico CONICET, La Plata, Provincia de Buenos Aires, Argentina

**Keywords:** *Bordetella pertussis*, outer-membrane vesicles, pertussis, combined vaccine, Th1, modulator, CD4+TRM cells

## Abstract

Acellular pertussis (aP) vaccines have markedly reduced the global burden of severe pertussis. However, their limited ability to elicit mucosal and durable immunity has been linked to waning protection and sustained *Bordetella pertussis* circulation. Selective pressure exerted by widespread aP vaccination has contributed to the emergence and regional dissemination of pertactin-deficient (PRN^−^) strains, raising additional concerns regarding vaccine effectiveness. In this context, we investigated whether incorporating outer membrane vesicles (OMVs) derived from *B. pertussis* into the aP vaccine could enhance its immunological profile, specifically by promoting Th1/Th17 polarization, inducing tissue-resident memory (TRM) T cells, and broadening protective coverage to include PRN^−^ isolates, while maintaining aP-induced immunity against lower respiratory tract colonization. Using a murine intranasal challenge model with a two-dose vaccination schedule, we assessed the safety, immunogenicity, and protective capacity of the OMV+aP vaccine prototype (combined) versus aP vaccine. The combined formulation was well tolerated and induced robust systemic and mucosal responses, characterized by higher IgG2a/IgG1 ratios, increased Th1/Th17 cytokine production (IFN-γ, IL - 17, and IL - 22), and elevated anti-*B. pertussis* IgA titers. Flow cytometric analyses revealed lung- and nasal-resident CD4^+^ TRM cells in the combined immunized mice, which were absent in those receiving aP alone. Functionally, OMV+aP formulation conferred superior protection in pulmonary and nasal compartments, significantly reducing lung bacterial loads (including against PRN^−^ strains) and uniquely diminishing nasal colonization even under high-dose challenge conditions. Passive transfer experiments confirmed the role of cellular and humoral immunity in bacterial clearance. These results demonstrate that OMVs synergize with aP to enhance immune response magnitude and quality, addressing key gaps in current aP vaccines and offering a next-generation strategy to prevent both disease and transmission.

## Introduction

Pertussis, a highly contagious respiratory disease caused by the Gram-negative bacterium *Bordetella pertussis*, remains a persistent and complex global health challenge, despite the availability of vaccines for over seven decades (1–4). While the disease can affect individuals of all ages, it is particularly severe in unvaccinated or under-vaccinated infants, often leading to hospitalization and, in many cases, death. Clinically characterized by paroxysmal coughing, cyanosis, and episodes of apnea, pertussis continues to impose a substantial burden on pediatric healthcare systems worldwide, including an increasing impact among older children and adolescents (5–10).

Global vaccination efforts using whole-cell (wP) and acellular (aP) pertussis vaccines have significantly reduced childhood morbidity and mortality. However, the resurgence of pertussis in recent decades, even in countries with consistently high aP vaccine coverage, such as the United States, the United Kingdom, and Australia, has raised major concerns about the effectiveness of current immunization strategies (1, 11, 12). Investigations into the causes of this resurgence suggest it is multifactorial, involving waning immunity (13, 14), pathogen evolution toward vaccine-resistant strains (15–20), particularly in the context of aP-induced immunity, and the limited capacity of existing vaccines to prevent nasopharyngeal colonization (21).

The COVID - 19 pandemic further exacerbated this epidemiologic situation by disrupting global immunization programs and eroding public trust in vaccines, contributing to dangerous declines in pertussis vaccine coverage (22, 23). According to WHO/UNICEF estimates, global coverage with the third dose of DTP (which includes pertussis) dropped from 86% in 2019 to 81% in 2021, with only partial recovery to 84% by 2023. Furthermore, the number of “zero-dose” children, those who received no vaccines at all, rose to 14.5 million in 2023, up from 12.9 million in 2019, with the highest impact observed in low- and middle-income countries. These trends represent a serious threat to global pertussis control and underscore the urgent need for more effective vaccines that provide durable immunity and enable simplified vaccination schedules (24–26).

Current pertussis vaccines include either whole-cell formulations, more reactogenic but immunologically robust, inducing mainly Th1/Th17 responses, or acellular formulations, which are safer but generate weaker and less durable immunity, skewed toward a Th2 profile (14, 24, 27, 28). Notably, aP vaccines are particularly ineffective at preventing nasal colonization, thereby allowing asymptomatic transmission (21). Moreover, the emergence and spread of pertactin-deficient (PRN^−^) *B. pertussis* strains prevalent in several region has further compromised aP vaccine effectiveness, as PRN remains a key antigen in most aP formulations (16, 29–31).

In response to these limitations, new strategies are being developed. One promising approach, which we explore in this study, involves the use of outer membrane vesicles (OMVs) derived from *B. pertussis*. OMVs naturally contain a wide array of immunogenic proteins and pathogen-associated molecular patterns (PAMPs) (32–35). Preclinical studies have shown that OMVs can elicit strong Th1/Th17 immune responses, significantly reduce bacterial loads in the lungs, and display lower reactogenicity compared to wP vaccines (31, 36–47). Intranasal administration has also been shown to reduce colonization in the upper respiratory tract (46, 48). In addition, OMVs possess intrinsic adjuvant properties, enhancing the immunogenicity of co-administered antigens and promoting a more protective immune profile (49, 50).

In this study, we evaluated the combination of OMVs with the commercial aP vaccine to assess whether this strategy could address the limitations of current aP vaccines and, importantly, facilitate future clinical evaluation through non-inferiority trials, given that the combined formulation includes components already licensed for human use. Specifically, we assessed whether the combined formulation could generate a more robust humoral immune response, modulate the aP-induced immune profile toward a protective Th1/Th17 bias, promote the induction of CD4+ TRM cell population, and enhance mucosal protection, including against nasal colonization. We also explored whether this approach could improve protection against strains more resistant to aP-induced immunity. To further characterize the quality of the induced immunity, we investigated immune cell memory recall using adoptive transfer models.

These efforts aim to provide a strong scientific foundation for the development of next-generation pertussis vaccines capable of inducing long-lasting immunity and restoring effective population-level protection.

## Materials and methods

### *B. pertussis* strains and growth conditions

*B. pertussis* gentamicin resistant strains used for challenge in the murine protection model included the reference Tohama phase I strain (CIP 8132, *ptxP1-ptxA2-prn1*), which expresses the adhesin pertactin (PRN), as well as clinical isolates (*ptxP3-ptxA1-prn2*) from the United States and Argentina that do not express the vaccine antigen pertactin (51–53). Bacteria were grown in Bordet–Gengou (BG) agar supplemented with 10% (v/v) defibrinated sheep blood (BGAS) and 50 μg/mL of gentamicin (BGAS-Gn)for 72 h at 36.5°C. Isolated colonies were replated in the same medium for 24 h and then resuspended in phosphate-buffered saline (PBS). The optical density at 650 nm was measured and serial 10-fold dilutions plated onto BG-blood agar to determine the number of bacteria in the challenge inoculum. To obtain OMVs, *B. pertussis* Tohama phase I strain CIP 8132, 24 h colonies grown in BGAS medium were used to seed liquid medium Stainer–Scholte (SS) (54).

### Isolation and characterization of OMVs

OMVs used for vaccine formulation were isolated and characterized as previously described (37, 55). Briefly, culture samples from the decelerating growth phase in SS medium were centrifuged and the bacterial pellet obtained was resuspended in 20 mM Tris–HCl, 2 mM EDTA pH 8.5. The suspension was sonicated in cool water for 20 min. After two centrifugations at 10,000×g for 20 min at 4°C, the supernatant was pelleted at 100,000×g for 2 h at 4°C. This pellet was re-suspended in Tris buffer (20 mM pH 7.6). The samples obtained were negatively stained for electron microscope examination. Protein content was estimated by the Bradford method using bovine serum albumin as standard (56). The presence of the main immunogenic proteins in the OMVs was detected by immunoblot assays using specific antibodies as we previously described (not shown) (37, 40).

### Combined OMV+aP vaccine composition

The vaccine formulation tested here combined 1/10 of the human dose of commercial ADACEL^®^ vaccine (aP, Sanofi Pasteur Limited) and 3µg of formalin detoxified OMVs derived from *B. pertussis* (hereafter named as combined). The pertussis component of commercial aP vaccine per human dose consists of 2.5 µg detoxified pertussis toxin (PTx), 5 µg filamentous hemagglutinin (FHA), 3 µg pertactin (PRN), 5 µg fimbriae types 2 and 3 (FIM).

### Mice immunization

**Figure 1:**
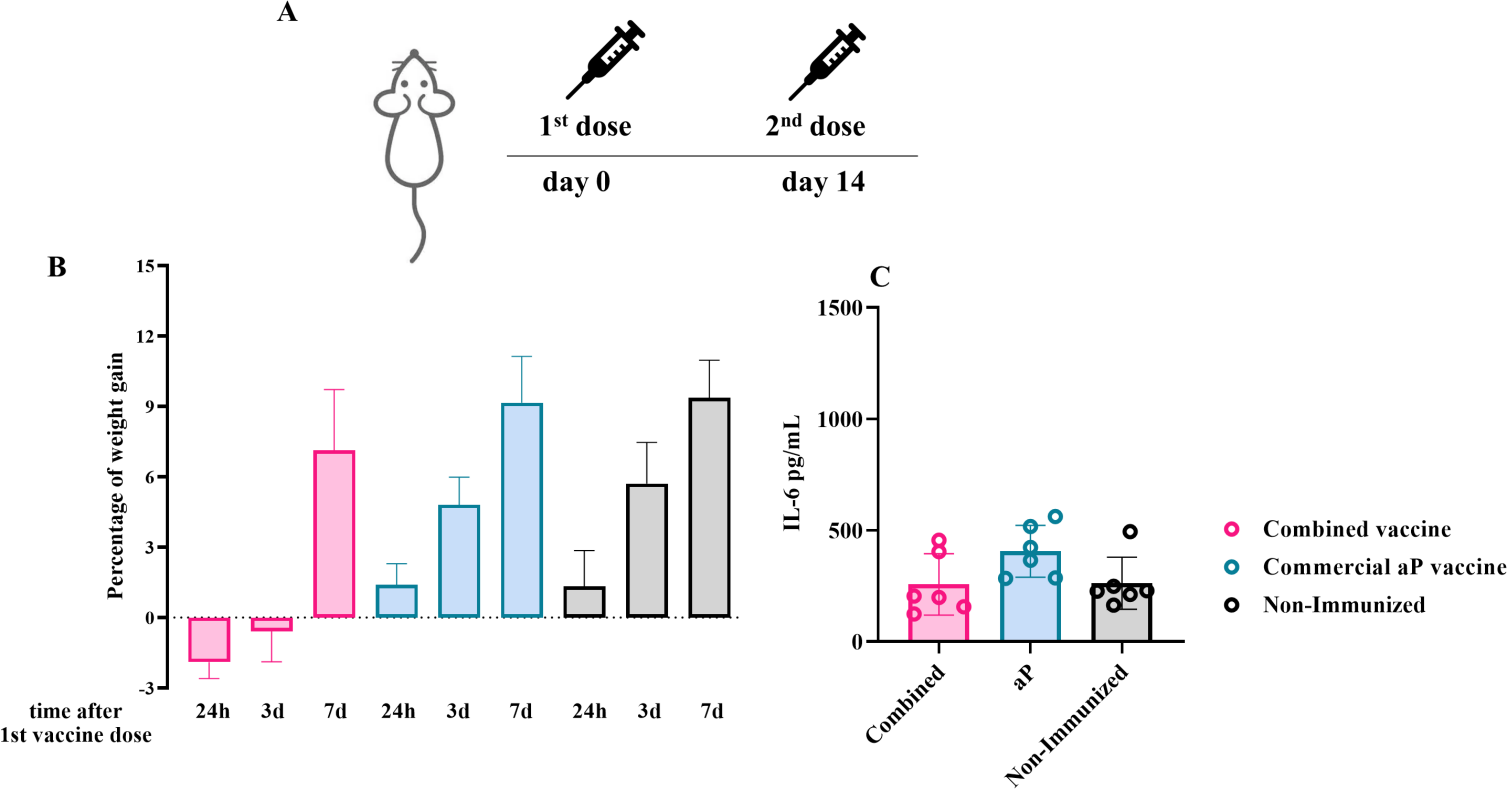
**(A)** Schematic representation of experimental vaccination schedule. BALB/c mice (n=6/group) were intramuscularly immunized at days 0 and 14, with a 2-dose scheme using the combined vaccine formulation and aP commercial vaccine. Non-immunized mice were incorporated as controls. **(B)** Mouse weight gain test. Animals were monitored for 7 days, with body weights recorded at 24 hours, 3 days, and 7 days post-intervention. The graph displays the percentage of weight gained by each animal relative to its initial baseline weight. No statistically significant differences in weight gain were observed between immunized and non-immunized groups in each time point tested (p > 0.05). **(C)** Inflammatory marker upon systemic vaccination. IL - 6 levels were measured by ELISA in sera obtained from mice 4h after the 1st vaccine dose. Bars represent the means ± SEM of pg/mL. No statistically significant differences in IL - 6 levels were observed among all studied groups (p > 0.05).

Animal experiments were performed using BALB/c mice (4–6 weeks old), provided by the Faculty of Veterinary Sciences, La Plata, Argentina. Animals were kept in ventilated cages and housed under standardized conditions with regulated daylight, humidity, and temperature. They received food and water *ad libitum*. The animal protocols were authorized by the Ethical Committee for Animal Experiments of the Faculty of Science at La Plata National University (approval number 004 - 06-15, 003 - 06–15 extended its validity until August 10, 2027). A two-dose intramuscular immunization schedule, with doses administered 14 days apart, was used to immunize groups of 4–6 BALB/c mice with either the combined OMV+aP vaccine or 1/10 of the human dose of the commercial aP vaccine ADACEL^®^ (**Figure 1A**). Non-immunized mice served as the control group.

### Mouse weight gain test

The MWG-test was carried out using groups of 6–8 BALB/c mice (14–16 g) which were vaccinated with the here tested vaccine formulation. Animals were observed for 7 days and body weight was recorded after 16 h, 3 and 7 days. Vaccines were considered non-toxic when passing the WHO and EP requirements (57).

### Expression of inflammatory marker upon systemic delivery of vaccines

In order to have an additional marker of pro-inflammatory capacity of the different formulations, serum was collected 4 h after each immunization by submandibular bleeding and serum separation. Serum IL - 6 was measured by ELISA using ELISA Flex: Mouse IL - 6 (HRP) Mabtech following manufacturer’s instructions.

### Stimulation of human whole blood

The whole blood IL - 6 cytokine release assay was performed as described by Stoddard et al. (58). Briefly, a 245 μl of blood sample was dispensed into each well of a 96-well tissue culture plate. Serial dilutions of each vaccine were prepared in RPMI 1640 cell culture medium (GIBCO) in a range of 5–300 ng/ml, incubated with the whole blood and then briefly centrifuged. A sample of 55 μl of plasma from each well was removed and frozen pending quantification of the cytokines. IL - 6 levels were measured by ELISA using BD OptiEIA (BD Biosciences) following manufacturer instructions.

### Intranasal challenge mouse model

For protection assays, mice were intranasally challenged with a sublethal dose of *B. pertussis* (as specified in the figure legends). Animals were euthanized one-week post-challenge, and bacterial loads in lungs and nasal tissue were quantified. Organs were aseptically excised, serially diluted, and plated for colony counting according to previously established protocols (31, 40, 48). Specifically, lungs and the nasal tissue was aseptically excised to minimize contamination from environmental bacteria.

Once extracted, the biological material was mechanically homogenized in a known volume of sterile phosphate-buffered saline (PBS). Subsequently, serial 10-fold dilutions of the homogenates were prepared and plated onto BGAS. The plates were incubated at 36.5°C for 72 hours. After incubation, colony-forming units (CFU) were counted to determine the bacterial load per lungs and nose. A minimum of three independent experiments were performed.

To study protection conferred by passive transfer, pooled serum (100 µl) or spleen cells (2.5 × 10^7^) derived from non-immunized or immunized mice were transferred intraocularly into 4-week-old naïve recipient mice. Twenty-four hours later, the mice were infected with *B. pertussis* and protection assessed on day 7 as described above.

### Enzyme-linked immunosorbent assay

Blood samples were collected two weeks after the final immunization and at sacrifice.

To detect antibody levels induced in mice by vaccination, Enzyme-linked immunosorbent assay (ELISA) was performed as we previously described (31, 40, 59). Plates (Nunc A/S, Roskilde, Denmark) were coated with sonicated *B. pertussis* Tohama phase I (whole cell lysates) or purified recombinant PTxA both at 3 µg/mL in 0.5 M carbonate buffer, pH 9.5 by overnight incubation at 4°C. Blocked plates with 3% milk in PBS (2 h 37°C) were incubated with serially diluted samples of mouse serum (1 h 37°C). Sera was obtained after leaving the blood samples to clot for 1 h at 37°C followed by centrifuging for 10 min at 6,000xg. Total IgG, IgG-isotypes and IgA from individual serum or pooled sera bound to the plates were detected after a 2-h incubation with goat anti–mouse-IgG–linked horseradish peroxidase (1:8,000 Invitrogen, USA) and goat anti–mouse-IgA–linked horseradish peroxidase (1:3,000) (Abcam). For measuring IgG isotypes, detection of bound antibody was determined using HRP labeled subclass-specific anti-mouse IgG1 (1:3,000), IgG2a (1:2,000) (Invitrogen USA). As substrate 1.0 mg/mL o-phenylendiamine (OPD, Bio Basic Canada Inc) in 0.1 M citrate-phosphate buffer, pH 5.0 containing 0.1% hydrogen peroxide was used. Optical densities (ODs) were measured with BioTek 800 TS microplate reader (BioTek, Agilent Technologies, US) at 490 nm. From the experimental protocol performed in triplicate, one representative experiment is presented in the Results.

### Avidity assay

Avidity was measured by an ELISA elution assay as the overall strength of binding between antibody and antigen, using plates incubated for 10 min with increasing concentration of ammonium thiocyanate (NH_4_SCN) from 0 to 1 M. Antibody avidity was defined as the amount (percentage) of antibody retained for each increment of NH_4_SCN concentration relative to that measured in absence of NH_4_SCN.

### Ag- specific IFN-γ, IL - 17, IL - 22 and IL - 5 production by spleen cells

After *B. pertussis* challenge, spleens from untreated and immunized mice were passed through a 40-mm cell strainer to obtain a single-cell suspension. Spleen cells were seeded in 48 well culture plates in a final volume of 500 µl/well RPMI 1640 with 10% fetal bovine serum, containing 100 IU/mL penicillin and 100 µg/mL streptomycin (44). All cell samples were stimulated with heat killed bacteria suspension 5x10^6^ UFC/well (HK_Bp_), PTx subunit A (PTxA1 μg/mL) or medium only. After 72 h of incubation (37 °C and 5% CO_2_), IFN-γ, IL - 17, IL - 22 and IL - 5 concentrations were quantified in supernatants by ELISA (Mabtech, USA) using conditions recommended by the manufacturer.

### FACS analysis of tissue resident memory CD4+T cells in lungs and nose

To discriminate blood-borne circulating cells from tissue-localized cells we administered intravenously anti-mouse PE-CD45 Ab (eBioscience) 10 min before they were euthanized as previously described (52). After the enzymatic disruption of tissue for 1 h at 37 °C with Collagenase D (1 mg/mL; Sigma-Aldrich) and DNAse I (20 U/mL; Sigma-Aldrich) and the lysis of red blood cells, cells from lungs and noses were incubated with CD16/CD32 FcgRIII (1:100) to block IgG Fc receptors. Cells were incubated with LIVE/DEAD Aqua (Invitrogen), followed by specific surface TRM-CD4+ T cells staining with CD45.2-BV650 (BD), CD3-PeCy7 (BD), CD4-FITC (Invitrogen), CD44-PE (BD), CD62L-PE-CF594 (BD), CD103-APC-EF780 (Invitrogen), CD69-APC (Invitrogen) (**Supplementary Figure S1**).

Fluorescence minus one or non-specific isotype Abs were used as controls. Flow cytometric analysis was performed on an LSR Fortessa, and data were acquired using Diva software (BD Biosciences). The results were analyzed using FlowJo software (TreeStar) (60).

### Statistical analysis

The data were evaluated statistically by t- Student test, two-way or one-way analysis of variance (ANOVA) followed by Bonferroni for multiple comparisons (via the GraphPad Prism^®^ software). Differences were considered significant at a p <0.05.

## Results

### Preclinical evaluation of the safety of a combined *B. pertussis* vaccine based on OMVs and commercial acellular formulation

Although it may seem straightforward to assume that vaccine combinations offer clear benefits for both individuals and public health systems, formulations that include multiple antigens must overcome unforeseen challenges, even when combining vaccines with well-established efficacy. Immunological, physical, and/or chemical interactions among the combined components may alter the host immune response (61). Therefore, a key challenge in the development of combination vaccines is the potential reduction in efficacy or safety compared to the individual components. Accordingly, our first experiment aimed to evaluate the safety of a combination of the commercial acellular vaccine and outer membrane vesicles derived from *B. pertussis* (hereafter referred to as combined) using a murine intranasal challenge model. Since the aim of this study is to address the limitations of the commercial acellular vaccine (aP) through a combined formulation including both aP and OMVs, all experiments presented herein were conducted in direct comparison with the aP vaccine. As a negative control, we included a group of animals that received no immunogens.

For the combined formulation, we used doses previously shown to be safe and immunogenic when administered individually in the same animal model. As in subsequent experiments, groups of BALB/c mice were immunized intramuscularly (i.m.) following a two-dose schedule with a 14-day interval between the first and second doses (**Figure 1A**).

Vaccine safety in the murine model was assessed by both monitoring body weight gain and measuring serum levels of the pro-inflammatory cytokine IL - 6 at 4 hours post-administration of the first dose (**Figures 1B, C**). For the weight gain test, animals were weighed at 24 h, 3 days, and 7 days post-immunization. As expected, all PBS-treated mice steadily gained weight at all time points analyzed.

While the acellular vaccine did not cause any reduction in weight at any of the assessed time points, the combined vaccine induced a slight, non-significant decrease in body weight at 24 and 72 hours. By day 7, mice in both vaccinated groups had recovered and showed more than 60% of the weight gain observed in the non-immunized control group.

Taken together, these results indicate that both the combined and acellular vaccine formulations meet the safety criteria based on the weight gain curve and can be considered non-toxic in this model.

Furthermore, serum IL - 6 levels, used as a marker of pro-inflammatory response, reached 257.0 ± 47.5 pg/mL at 4 hours post-immunization with the combined vaccine. A similar level was observed with the commercial aP vaccine used at 1/10 of the human dose (405.7 ± 47.5 pg/mL). Importantly, both values were not significantly different from those measured in the untreated control group. Additionally, in *in vitro* human whole-blood assays, we observed that IL - 6 levels induced by OMV doses ranging from 0.25 to 3 µg (combined with 1/10 of the human aP dose) ranged from 2 ng/mL to 12 ng/mL for the lowest and highest OMV quantities tested. These levels were lower than those induced by an approved commercial whole-cell vaccine (~60 ng/mL). No cytokine stimulating activity was detected for the commercial aP vaccine. Moreover, no visible signs of local reactogenicity at the injection site, such as erythema, swelling, or induration, were observed in any of the vaccinated animals. This visual assessment, performed by trained personnel, further supports the safety profile of both the combined and aP formulations in this model.

### Immunogenicity of combined vaccine

Given the known properties of OMVs to induce Th1, Th2, and Th17 immune profiles (31, 43, 44, 46, 62), as well as their adjuvant and immunomodulatory capabilities (49), we evaluated the humoral immune response specific to pertussis toxin (in particular PTxA) and a heat-killed *B. pertussis* lysate (HK_Bp_). Specifically, we comparatively assessed the levels of IgG and the IgG1 and IgG2a isotypes specific to these antigens in response to the combined vaccine formulation and the commercial aP vaccine.

**Figure 2:**
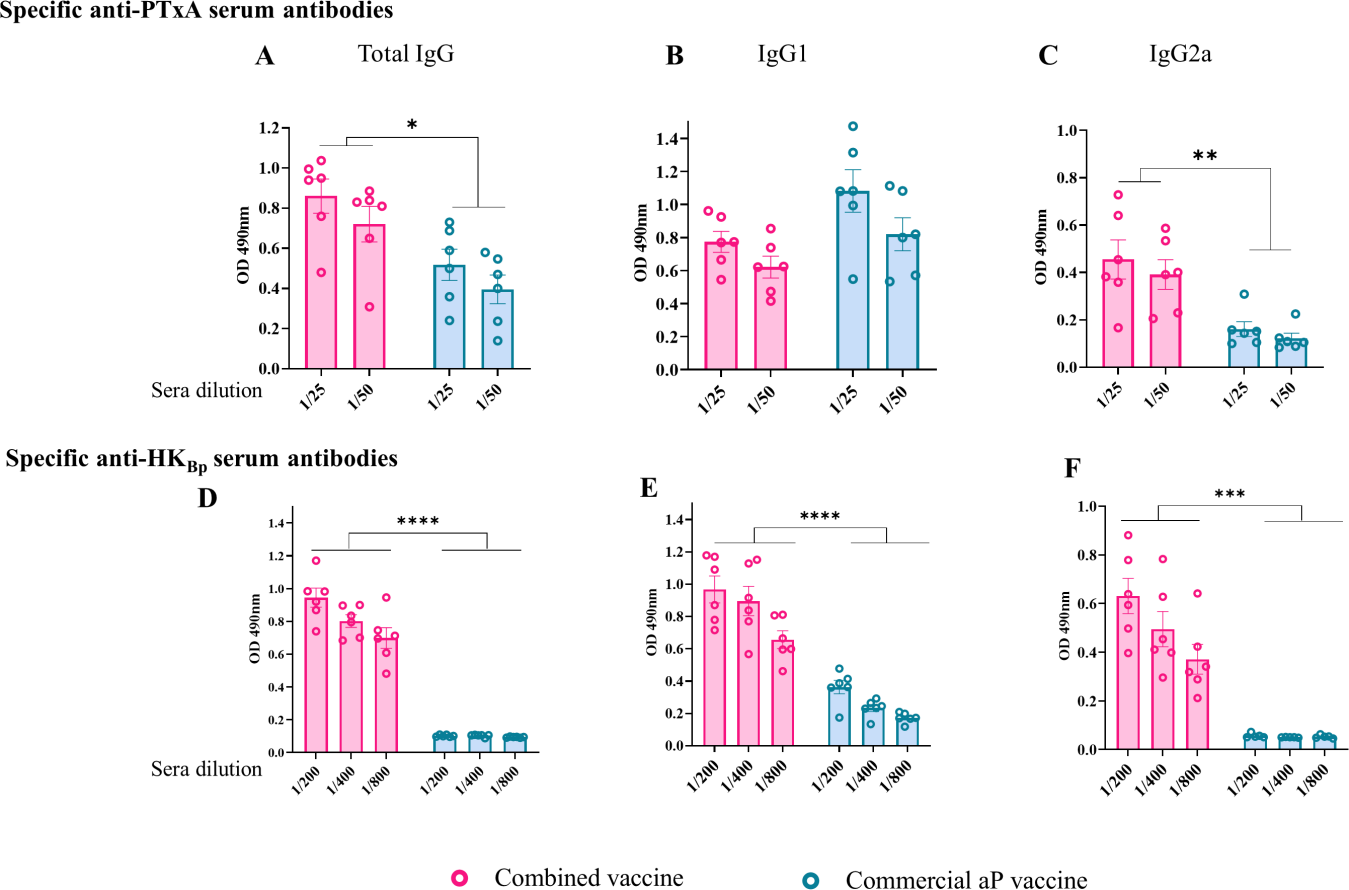
Characterization of the humoral immune response induced by the combined vaccine (OMV_Bp_+aP). Antibody responses specific to pertussis toxin (PTxA; panels **A–C**) and heat-killed *B*. *pertussis* lysate (HK_Bp_; panels **D–F**) were evaluated by ELISA in sera collected 14 days after the second vaccine dose. Absorbance values at 490 nm were measured for two or three serum dilutions. Sera from mice immunized with the combined vaccine were compared to those receiving the aP vaccine or non-immunized controls. Antigen-specific levels of total IgG **(A, D)**, IgG1 **(B, E)**, and IgG2a **(C, F)** are shown. Statistically significant differences are indicated (*p < 0.05, **p<0.01, ***p<0.001, ****p<0.0001).

Using the previously described two-dose immunization schedule (**Figure 1A**), triplicate experiments demonstrated that the combined formulations elicited a more robust antibody response (**Figure 2**). Notably, mice immunized with the combined vaccine exhibited significantly higher serum IgG antibody titers against both PTxA (p < 0.05) and HK_Bp_ (p < 0.0001) compared to those receiving the aP treatment alone. As expected, IgG levels in unimmunized mice were markedly lower (data not shown). The NH_4_SCN avidity assay using the chaotropic agent showed that the antibodies induced by the combined vaccine (anti-PTxA IgG) were of slightly higher quality than those elicited by the aP vaccine, although the difference was not statistically significant (not shown).

Regarding IgG isotype profiles, mice immunized with the aP vaccine exhibited marginally higher (though statistically insignificant) levels of PTxA-specific IgG1 compared to those receiving the combined formulation, while PTxA-specific IgG2a levels were significantly lower (p < 0.05). This pattern aligns with the established Th2-skewed response characteristic of aP vaccines, whereas the combined vaccine preferentially induced a Th1-biased response. For HK_Bp_-specific antibodies, both IgG1 and IgG2a titers were significantly reduced in aP-vaccinated mice relative to the combined vaccine group (p< 0.001). Notably, the combined formulation elicited slightly higher IgG2a than IgG1 levels against HK_Bp_, further supporting its Th1-polarizing capacity (**Figures 2E, F**).

These results are of particular interest as they demonstrate that the addition of OMVs to the aP vaccine enhances the humoral immune response and modulates the immune profile toward Th1 polarization.

**Figure 3:**
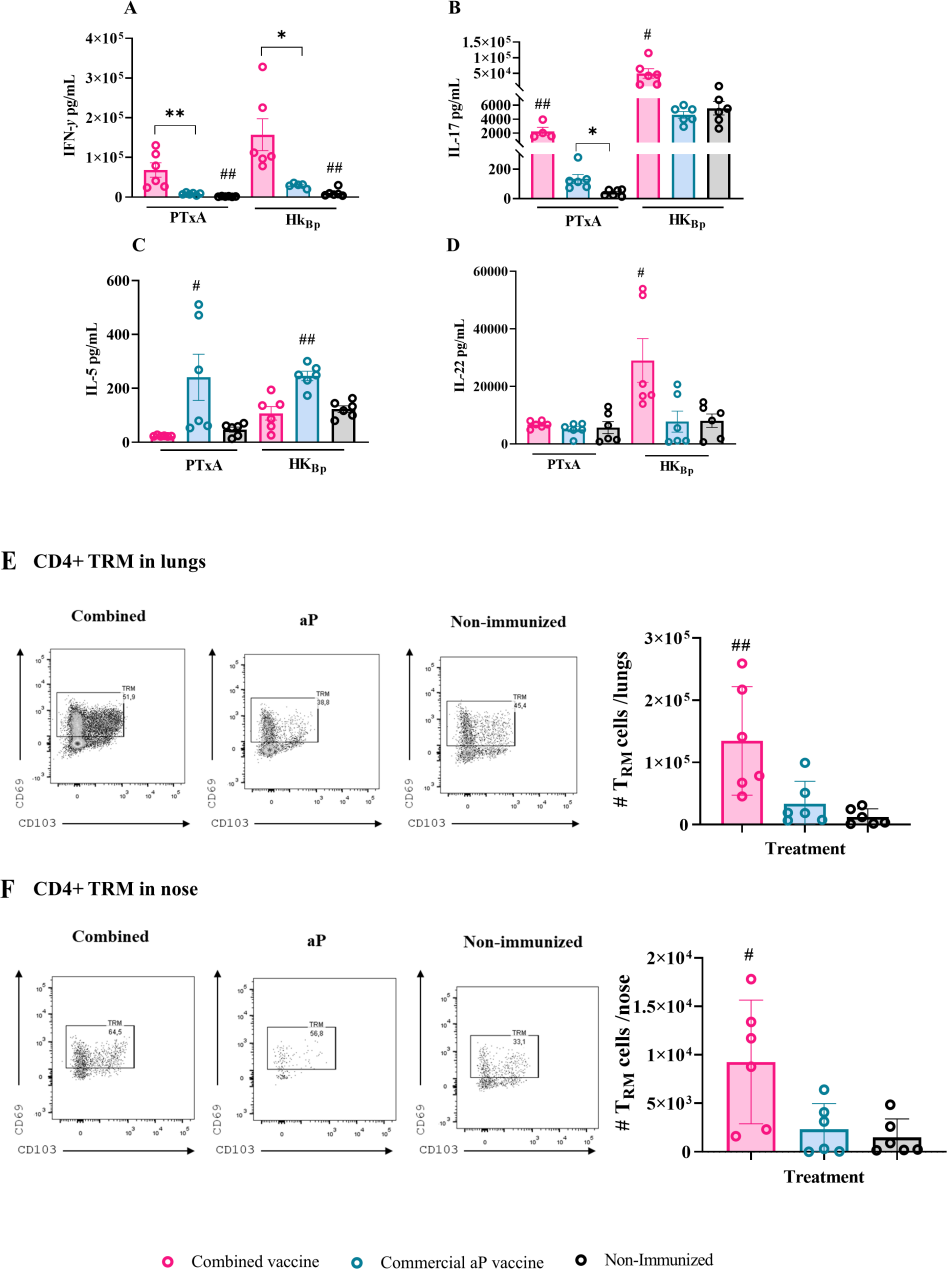
Cellular immune response induced by the combined vaccine formulation. Mice were immunized intramuscularly on Days 0 and 14 with the combined formulation (OMV+aP) as described in **Figure 1**. Seven days after challenge with a sublethal intranasal dose of *B*. *pertussis* (1×10^7^–5×10^7^ CFU in 40 µL), mice were euthanized, and spleens were harvested. Splenocytes were restimulated *ex vivo* with heat-killed *B*. *pertussis* (HKBp; 5×10^6^ CFU/well), pertussis toxin subunit A (PTxA; 1 µg/mL), or medium only (non-stimulated, not shown for simplicity of the figures). Cytokine concentrations of IFN-γ **(A)**, IL - 17 **(B)**, IL - 5 **(C)**, and IL - 22 **(D)** in culture supernatants were measured by ELISA. Bars represent mean ± SEM (pg/mL). Absolute counts of CD4+ TRM (CD45–, CD44+, CD62L−, CD69+, CD103+/–, CD4+) in lungs and nose are represented in panels **(E, F)** respectively. Bars represent mean ± SEM CD4+TRM cells count/lungs or nose. Statistical analysis was performed by two-way ANOVA followed by Bonferroni *post hoc* test. * and ** indicate significant pairwise differences (*p < 0.05, **p < 0.01). # and ## indicate significant differences between the treatment and the other groups (#p<0.05, ##p < 0.01).

To further investigate the ability of OMVs to modulate the immune response of combined vaccine formulations toward a Th1 profile, we performed spleen cell stimulation assays using splenocytes from mice immunized with different vaccine formulations, as well as from non-immunized controls (**Figure 3**). The stimuli used were the A subunit of pertussis toxin (PTxA) and the HK_Bp_. After the incubation period, we evaluated IFN-γ levels as a marker of Th1-type response (**Figure 3A**). Additionally, we assessed IL - 17 [Th17 profile marker, **Figure 3B**)] and IL - 5 (Th2 profile marker, **Figure 3C**) levels to further characterize the immune response induced by the tested combined formulations.

The results were obtained from determinations performed after bacterial intranasal challenge, in alignment with the 3Rs principle, as the same challenged animals were used to evaluate protection against colonization.

**Figure 3A** shows that, for both stimuli, the presence of OMVs in the combined vaccines induced significantly higher IFN-γ levels compared to the aP formulation (68,415 ± 18,244 pg/mL vs. 8,217 ± 1,129 pg/mL, p<0.001 for PTxA stimulation; 157,291 ± 40,285 pg/mL vs. 29,610 ± 2,581 pg/mL for HK_Bp_ stimulation, p<0.05 between combined and aP, respectively). Similarly, IL - 17 levels (**Figure 3B**) were significantly higher in samples from mice immunized with the combined vaccine compared to those from mice vaccinated with aP (2,260 ± 568.8 pg/mL vs. 132.8 ± 30.67 pg/mL, *p* < 0.001 for PTxA stimulation; 49,772 ± 15,278 pg/mL vs. 4,625 ± 475.2 pg/mL for HK_Bp_ stimulation, *p* < 0.05).

In contrast, IL - 5 levels (**Figure 3C**) were significantly elevated in the aP group relative to the combined vaccine group (*p* < 0.001 for PTxA stimulation and *p* < 0.05 for HK_Bp_ stimulation).

We also measured IL - 22 levels (**Figure 3D**), given its recognized role as a key mediator of early mucosal defense and protection against epithelial lung damage (49, 50). Consistently, IL - 22 levels in samples from mice immunized with the combined vaccine were significantly higher than those from the aP group following HK_Bp_ stimulation (25,982 ± 7,104 pg/mL vs. 8,391 ± 3,153 pg/mL, *p* < 0.05). Notably, IL - 22 levels in aP-immunized mice were not significantly different from those in non-immunized controls. When the spleen cells were stimulated with PTxA, no differences were detected among the studied treatment.

Flow cytometry analysis revealed a significantly higher number of lung- (**Figure 3E**) and nasal-resident (**Figure 3F**) memory CD4^+^ T cells in OMV+aP-treated mice compared to those that received the aP vaccine alone. These findings indicate that the inclusion of OMVs in the aP formulation enhances the recruitment and establishment of memory T cells in the respiratory mucosa, a response not achieved with the aP vaccine alone.

Altogether, these results demonstrate that the addition of OMVs to combined vaccine formulation is advantageous over the standalone aP formulation, not only in terms of enhancing the humoral response but also by promoting a Th1/Th17/Th22 immune profile, increasing epitope diversity due to their complex composition and resident memory CD4^+^ T cells.

### Protective capacity of the combined vaccine prototype against lung bacterial colonization (severe disease)

**Figure 4:**
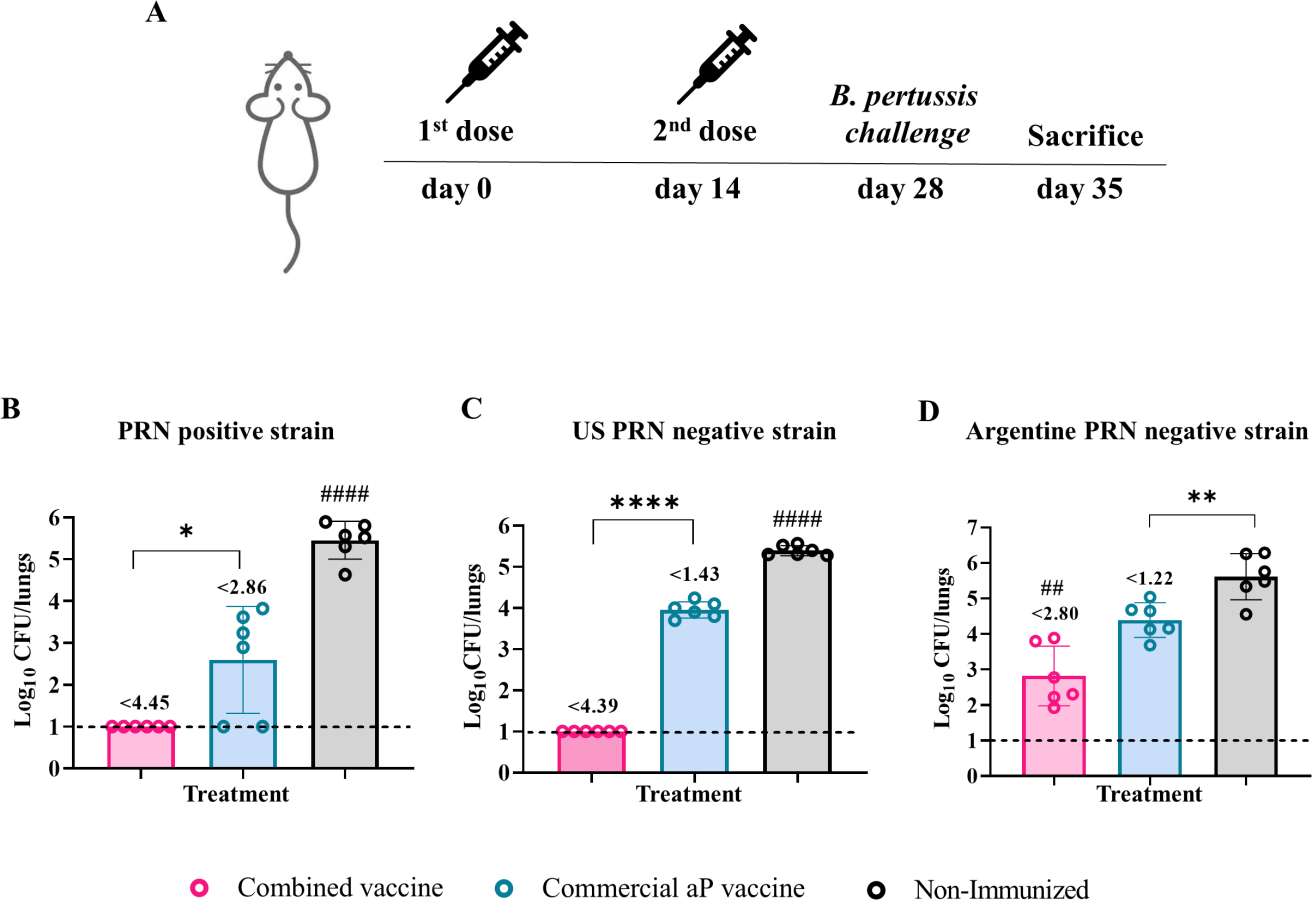
Protective capacity of the combined vaccine against PRN-positive and PRN-deficient *B*. *pertussis* strains. Mice were immunized on Days 0 and 14 with two doses of the indicated vaccine formulations and challenged intranasally on Day 28 with different *B*. *pertussis* strains **(A)**: PRN-positive reference strain (*B. pertussis* Tohama pahase I) **(B)**, PRN-deficient clinical isolate from the U.S. outbreak **(C)**, and locally circulating PRN-deficient clinical isolate **(D)**. Bacterial burden in the lungs was assessed 7 days post-challenge (Day 35). Results are expressed as log_10_ CFU per lungs. Numbers above the bars indicate the difference between the mean bacterial loads of each group and the non-immunized group, expressed in log_10_. Bars represent mean ± SEM. Statistical comparisons were performed using one-way ANOVA with Bonferroni correction. *, **, and **** indicate significant pairwise differences (*p<0.05, **p<0.01, ****p<0.0001). ## and #### indicate significant differences between the treatment and the other groups (##p<0.01, ####p<0.0001).

To evaluate the protective capacity of the different vaccine formulations tested in this study, groups of mice immunized with two-dose schedule were intranasally challenged with a sublethal dose of *B. pertussis* (1×10^7^–5×10^7^ CFU) 14 days after receiving the final dose (**Figure 4A**). Seven days post-challenge, all animals, including those in the non-immunized control group, were euthanized by cervical dislocation to assess bacterial colonization in the upper and lower respiratory tracts.

The challenge was performed using *B. pertussis* strains with distinct genotypic and phenotypic characteristics. These included the reference Tohama I phase I strain (*ptxP1-ptxA2-prn1*), which expresses the adhesin pertactin (PRN), and two *ptxP3-ptxA1-prn2* strains that lack PRN expression. One of the PRN-deficient strains (PRN^-^) corresponds to a locally circulating isolate, while the other derives from the 2012 U.S. outbreak, kindly provided by Dr. Lucia Tondella (CDC).

As expected, the non-immunized control group exhibited the highest bacterial burden in the lungs (3.02×10^5^ CFU/lungs, **Figure 4B**). Immunized groups showed significant reductions in lung colonization by the Tohama strain compared to controls (p < 0.0001). The highest reduction was observed in animals immunized with the combined OMV+aP formulation, which exhibited a 4.45-log decrease (complete bacterial clearance, **Figure 4B**). In contrast, mice immunized with the aP vaccine alone showed a 2.86-log_10_ reduction (**Figure 4B**).

When challenged with the U.S. PRN^−^ strain, mice receiving the combined vaccine showed complete bacterial clearance from the lungs (4.39-log decrease, p< 0.0001 **Figure 4C**). In contrast, aP-immunized mice exhibited bacterial loads close to those of the non-immunized group (1.43-log decrease, **Figure 4C**). A similar trend was observed with the locally circulating PRN^−^ strain (**Figure 4D**), though all groups showed higher residual bacterial loads compared to those seen with the U.S. PRN^−^ strain, suggesting intrinsic differences in virulence or immune evasion capacity between the PRN-deficient isolates.

### Passive transfer experiments

To dissect the respective contributions of humoral and cellular responses induced by the combined OMV+aP vaccine, passive transfer experiments were conducted using sera or splenocytes from immunized donor mice. Naïve recipient mice were subsequently challenged intranasally with *B. pertussis* Tohama phase I strain, and bacterial burden, antibody titers, and immune cell populations were assessed.

**Figure 5:**
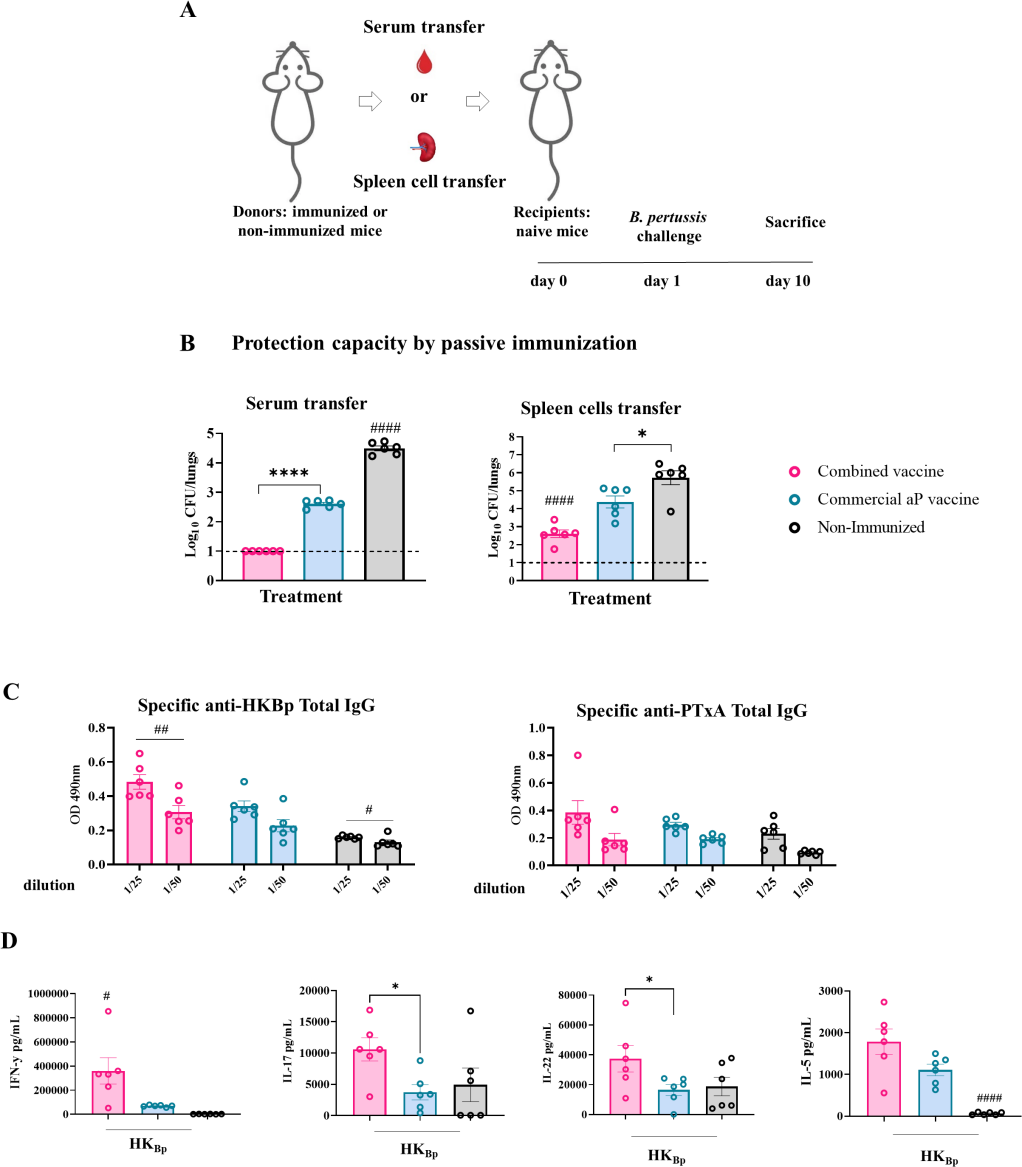
Protection capacity of passive transfer of serum or splenocytes from immunized mice (donors). **(A)** Experimental design: naïve recipient mice received either serum or splenocytes from donor mice immunized with 2-dose scheme with OMV+aP, aP, or PBS. One day later, recipients were challenged intranasally with a sublethal dose of (5x10*^7^* CFU) *B*. *pertussis* Tohama phase I strain, and bacterial burden in the lungs was quantified 10 days post-transfer. **(B)** Lungs bacterial load following either serum- or splenocyte- transfer. **(C)** IgG titers against HK_Bp_ and PTxA were measured in sera from recipient mice 7 days post-challenge. **(D)** Cellular immune response in receptor mice that received immune splenocytes cells. Seven days after challenge with a sublethal intranasal dose of *B*. *pertussis* (1×10^7^–5×10^7^ CFU in 40 µL), mice were euthanized, and spleens were harvested. Splenocytes were restimulated *ex vivo* with heat-killed *B*. *pertussis* (HKBp; 5×10^6^ CFU/well) or medium only (non-stimulated, not shown for simplicity of the figures). Cytokine concentrations of IFN-γ, IL - 17, IL - 5, and IL - 22 in culture supernatants were measured by ELISA. Bars represent mean ± SEM (pg/mL). Statistical analysis was conducted using one-way ANOVA with *post hoc* testing. *p<0.05, ****p<0.0001, #p<0.05, ##p<0.01, and ####p<0.0001.

Transfer of sera (**Figure 5A**) from OMV+aP-immunized mice conferred significantly higher protection in the lungs compared to sera from aP-immunized or PBS-treated donors (**Figure 5B**, p<0.0001). Lung bacterial counts were undetectable in recipients of sera from OMV+aP-immunized mice, indicating complete protection. In contrast, mice receiving sera from aP-vaccinated donors showed residual bacterial loads in the lungs (4.1 10^2^CFU/lungs), while PBS controls exhibited the highest bacterial burden (3.1 10^4^CFU/lungs).

Transfer of splenocytes (**Figure 5A**) from OMV+aP-vaccinated donors resulted in enhanced protection in the lungs colonization (3.1 -log decrease in comparison with control group, p< 0.0001) compared to recipients of aP- (1.3-log decrease in comparison with control group, p< 0.01) or PBS- splenocytes (**Figure 5B**). Mice receiving aP splenocytes exhibited only partial protection.

Consistently, sera from mice that received OMV+aP-derived splenocytes showed significantly elevated anti-*B. pertussis* IgG titers 7 days post-challenge, as determined by ELISA (p < 0.01), indicating the ability of transferred lymphocytes to support antibody production upon infection (**Figure 5C**). We also observed that post-challenge, recipients of immune cells from donor animals vaccinated with the combined vaccine showed significantly higher IFN-γ levels compared to those receiving cells from the aP-vaccinated group (359,802 ± 133,612 pg/mL vs. 69,517± 7,314 pg/mL; p<0.05 between combined and aP groups, respectively) (**Figure 5D**). Similarly, IL - 17 and IL - 22 levels were significantly elevated in mice that received splenocytes from combined-vaccinated animals versus those receiving splenocytes from aP-vaccinated animals (p<0.05 **Figure 5D**). No significant differences were observed for IL - 5.

Collectively, these data highlight the immunological superiority of the OMV+aP vaccine, which elicits robust humoral and cellular responses and confers enhanced protection not only against PRN^+^ strains but also against PRN^−^ isolates. Passive transfer studies support a mechanistic role for both antibodies and antigen-experienced lymphocytes in mediating protection, reinforcing the rationale for combined vaccine strategies.

### The combined vaccine reduces bacterial colonization in the upper respiratory tract, a key factor in pertussis transmission

Reducing the transmission of *B. pertussis* has long been a central goal of vaccination strategies, particularly given the extremely high contagiousness of the pathogen. The basic reproduction number (R_0_) for *B. pertussis* has been estimated at approximately 17, making it one of the most transmissible bacterial infections.

**Figure 6:**
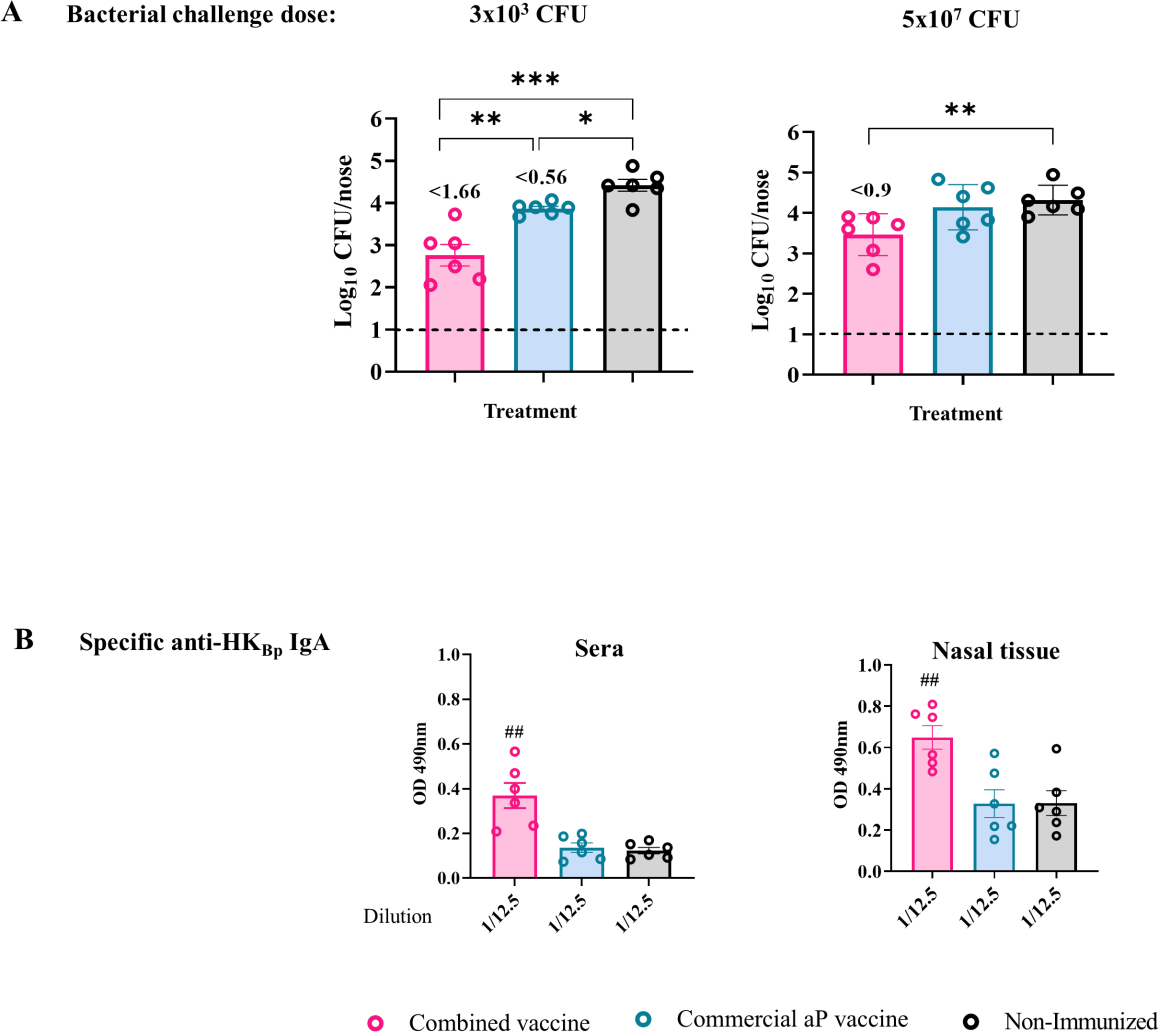
Protective capacity induced by the combined vaccine formulation against upper respiratory tract bacterial colonization. Mice were immunized on Days 0 and 14, then challenged intranasally with either a high dose (5×10^7^ CFU in 40 µL) or a low dose (3×10³ CFU in 6 µL) of *B*. *pertussis* Tohama phase I in volumes targeting the lungs and nose or only nasal cavity, respectively. Nasal bacterial load was evaluated 7 days post-challenge **(A)**. Numbers above the bars indicate the difference between the mean bacterial loads of each group and the non-immunized group, expressed in log_10_. Serum and nasal tissue IgA titers against HK_Bp_ were measured by ELISA at the same time point **(B)**. Bars represent mean ± SEM. Statistical analysis was performed using one-way ANOVA. *p<0.05, **p<0.001, ***p<0.0001, ##p<0.01.

Pioneering studies by Warfel et al. (21) using the non-human primate baboon model demonstrated that acellular pertussis vaccines, while effective at protecting against disease, fail to reduce bacterial colonization in the upper respiratory tract (URT), thereby allowing continued transmission despite immunization. This finding underscored the need to develop vaccines capable of inducing mucosal immunity and limiting nasopharyngeal colonization. Here we investigated whether the addition of OMVs to the aP vaccine formulation could enhance its ability to reduce *B. pertussis* colonization in the nasal cavity. Mice were immunized with a two-dose schedule, as previously described, and then challenged intranasally with the *B. pertussis* Tohama phase I strain using two different inoculum doses (**Figure 6A**):

1. A low dose (3 × 10³ CFU) delivered in a reduced volume to restrict bacterial colonization primarily to the nasal cavity.
2. A high dose (5 × 10^7^ CFU) administered in a larger volume to ensure the bacteria also reached the lower respiratory tract.

The high-dose challenge was included to assess the extent to which the treatments could reduce nasal colonization.

In non-immunized control animals, both doses resulted in comparable levels of colonization in the nasal cavity (~2.24 × 10^4^ CFU/nose). This finding suggests a local niche limitation that constrains bacterial growth in the URT regardless of the inoculum size. Mice immunized with the aP vaccine alone showed practically no reduction in nasal colonization under either challenge condition. In contrast, the combined OMV+aP vaccine demonstrated significant protective efficacy in the URT, with bacterial reductions of 1.66 logs (p < 0.001) and 0.9 log (p < 0.001) following challenge with 3 × 10³ CFU and 5 × 10^7^ CFU, respectively. These results indicate that the inclusion of OMVs may overcome the limitations of aP vaccines in controlling colonization and, by extension, transmission.

Given the established role of mucosal IgA in preventing colonization by *B. pertussis*, particularly in the nasal epithelium (63), we evaluated serum IgA levels post-vaccination. Elevated levels of antigen-specific IgA were observed in mice immunized with the combined formulation (**Figure 6B**, p< 0.001), both in sera and in nasal tissue homogenates, consistent with the improved control of bacterial colonization.

Together, these findings support the hypothesis that adding OMVs to aP vaccines enhances mucosal immunity, promoting IgA production and reducing colonization in the URT, a critical step toward curbing transmission of pertussis.

## Discussion

Current acellular pertussis (aP) vaccines, while significantly safer than first-generation whole-cell (wP) formulations, exhibit critical immunological limitations (64, 65). They predominantly elicit Th2-skewed responses, induce short-lived immunity, and fail to prevent bacterial colonization of the upper respiratory tract (URT), thereby allowing asymptomatic transmission (21, 27, 28). The emergence and global spread of pertactin-deficient (PRN^−^) *B. pertussis* strains have further undermined the protective capacity of conventional aP vaccines (16, 51, 52). PRN is a key antigen in most licensed formulations, and the expansion of PRN^−^ variants seem to reduce aP effectiveness (15, 66). However, post-pandemic surveillance has revealed heterogeneous trends: in some countries PRN^−^ strains have markedly declined or even ceased to circulate, while in others, particularly certain regions of the Americas and Europe, they continue to predominate. This dynamic landscape underscores the need for next-generation vaccines that confer broader and more durable protection against both classical and immune-evasive *B. pertussis* lineages. Outer membrane vesicles (OMVs) derived from *B. pertussis* represent a compelling platform to address these shortcomings. OMVs inherently package multiple immunogenic proteins and pathogen-associated molecular patterns (PAMPs), providing intrinsic adjuvanticity that promotes robust Th1 and Th17 immunity (31, 46–49). Preclinical studies, including our own prior work, have consistently demonstrated that OMV-based vaccines can significantly reduce bacterial loads in the lungs, induce mucosal IgA, and exhibit markedly lower reactogenicity than wP (37, 40, 43, 46–48, 67). Importantly, intranasal OMV administration has been associated with URT colonization control, a key feature absent from standard aP vaccination (21, 65, 68, 69).

In this study, we leveraged these immunological attributes by combining *B. pertussis*-derived OMVs with a licensed aP vaccine to generate a combined formulation aimed at overcoming the two principal limitations of current aP vaccines: poor mucosal immunity and vulnerability to immune-evasive bacteria (28). The combined OMV+aP vaccine demonstrated an excellent safety profile across *in vitro* and *in vivo* models, with no signs of local or systemic toxicity even at the highest OMV dose tested (3 µg in combination with 1/10 of the human aP dose). Human whole-blood assays confirmed low cytokine induction relative to wP, supporting the translational potential of this approach (43).

Functionally, the combined vaccine reshaped the humoral immune response compared to the aP vaccine alone. Mice immunized with OMV+aP generated higher total IgG titers against PTxA and heat-killed *B. pertussis* lysate (HK_Bp_) than those receiving aP alone. Isotype profiling revealed a shift toward a Th1-biased IgG2a response, both against purified antigens and whole-cell lysates, suggesting an enhanced potential for opsonophagocytosis and bacterial clearance.

Concomitantly, splenocytes from OMV+aP-vaccinated animals secreted markedly higher levels of IFN-γ and IL-17 following *ex vivo* antigenic stimulation, with the addition of a distinct IL-22 signal, a cytokine linked to mucosal barrier protection and epithelial antimicrobial responses (70, 71). Notably, IL-5 production remained unaltered, underscoring the transition from a Th2-dominated profile toward a functionally protective Th1/Th17 axis. These immune signatures translated into robust *in vivo* protection. The OMV + aP vaccine markedly reduced bacterial burden in both the lungs and the nasal cavity. In the lungs, colonization by the PRN^+^ strain (Tohama phase I) was decreased by 4.45 log_10_ CFU compared with non-immunized controls, surpassing the 2.86 log_10_ reduction achieved by the aP vaccine alone. In the nasal cavity, the combined formulation achieved a 1.66 log_10_ CFU reduction under low-dose challenge and a 0.9 log_10_ CFU reduction under the stringent high-dose model. Together, these results highlight the superior ability of the OMV + aP vaccine to limit both pulmonary infection and upper-airway colonization, addressing key limitations of current acellular vaccines. The ability to limit URT colonization is particularly noteworthy, as it directly addresses the Achilles’ heel of current aP vaccines: their inability to prevent transmission. Consistent with this mucosal effect, OMV+aP induced CD4^+^ tissue-resident memory (TRM) cells in both lungs and nasal mucosa, a cellular compartment that has emerged as a key correlate of near-sterilizing immunity against *B. pertussis* (24, 25, 72). The parallel increase in serum and nasal tissue IgA levels, which play a crucial role in URT protection, further reinforces the functional complementarity of humoral and cellular immunity elicited by this combined formulation (63, 73).

The breadth of protection was underscored by challenge experiments using PRN^−^ clinical isolates. Whereas aP vaccination alone conferred minimal protection, OMV+aP immunization effectively eliminated or markedly reduced lung colonization by both the U.S. outbreak PRN^−^ strain and a locally circulating Argentinean isolate. These findings underscore the combined vaccine’s ability to overcome antigenic drift and selective immune evasion, a critical feature for achieving long-term, population-level impact.

Passive transfer experiments provided mechanistic validation of the dual contribution of humoral and cellular immunity. Serum and splenocytes from OMV+aP-immunized donors conferred superior protection in naïve recipients, with sera achieving complete lung clearance of *B. pertussis* Tohama phase I strain and splenocyte transfer mediating profound reductions in bacterial burden (3.1-log decrease versus controls). Post-challenge analysis of cytokine production in recipient mice confirmed the transfer of Th1/Th17 functionality, including elevated IFN-γ, IL-17, and IL-22, linking these immune signatures to the observed protection.

Altogether, our data establish that OMV inclusion not only preserves the immunogenic properties previously described for this platform but also enhances the magnitude, quality, and mucosal reach of the immune response when combined with a licensed aP formulation. This strategy effectively addresses critical shortcomings of current aP vaccines, namely, weak mucosal immunity, limited durability, and susceptibility to PRN^−^ variants. Importantly, the use of clinically approved aP components provides a regulatory bridge for non-inferiority trial designs, offering a practical and accelerated path toward clinical translation.

In conclusion, the OMV+aP vaccine represents a next-generation pertussis candidate that is safe, highly immunogenic, and functionally superior to existing aP formulations. By simultaneously eliciting potent systemic and mucosal immunity, including TRM induction and IL-22–mediated mucosal defense, this strategy offers a feasible and strategically sound approach to strengthen global pertussis control and reduce transmission.

## Data Availability

The original contributions presented in the study are included in the article/**Supplementary Material**. Further inquiries can be directed to the corresponding author.

## Supplementary material

Supplementary Figure 1 Gating strategy for the flow cytometric analysis of tissue-resident memory (TRM) CD4^+^ T cells in lungs and nasal tissue. To discriminate tissue-localized lymphocytes from circulating cells, mice received an intravenous injection of PE-conjugated anti-mouse CD45 antibody (eBioscience) 10 min prior to euthanasia, as previously described (60). Lungs and nasal tissues were enzymatically digested for 1 h at 37 °C using Collagenase D (1 mg/mL; Sigma-Aldrich) and DNase I (20 U/mL; Sigma-Aldrich). Following red blood cell lysis, single-cell suspensions were incubated with CD16/CD32 FcγRIII (1:100) to block Fc receptors, stained with LIVE/DEAD Aqua (Invitrogen), and subsequently labeled with the following antibodies: CD45.2-BV650 (BD), CD3-PeCy7 (BD), CD4-FITC (Invitrogen), CD44-PE (BD), CD62L-PE-CF594 (BD), CD103-APC-EF780 (Invitrogen), and CD69-APC (Invitrogen). TRM CD4^+^ T cells were defined as CD45^−^ CD44^+^ CD62L^−^ CD69^+^ CD103^+^/^−^ CD4^+^. Fluorescence minus one (FMO) and isotype control antibodies were used to validate gating. Flow cytometric acquisition was performed on an LSR Fortessa using Diva software (BD Biosciences), and data were analyzed with FlowJo (TreeStar).
