# Supplementary figures and images for: Harnessing outer membrane vesicles derived from Bordetella pertussis to overcome key limitations of acellular pertussis vaccines
Source: Front Immunol. 2025 Sep 2;16:1655910. doi: 10.3389/fimmu.2025.1655910 (PMC12436301; doi:10.3389/fimmu.2025.1655910)

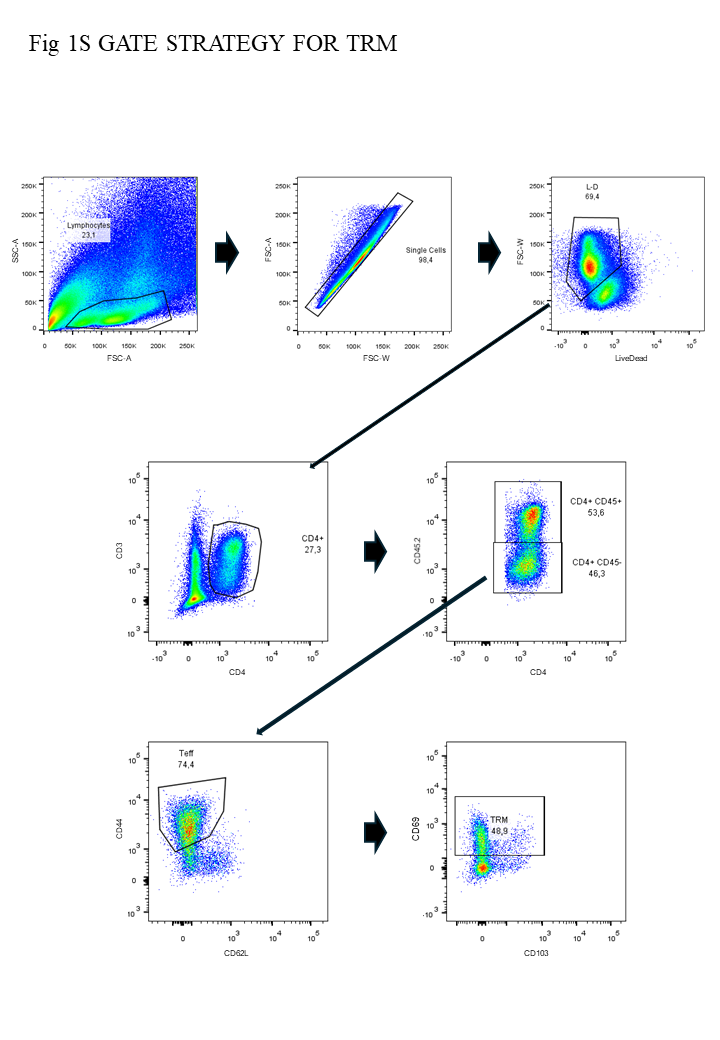

Supplement: Supplementary Figure 1 — Gating strategy for the flow cytometric analysis of tissue-resident memory (TRM) CD4+ T cells in lungs and nasal tissue. To discriminate tissue-localized lymphocytes from circulating cells, mice received an intravenous injection of PE-conjugated anti-mouse CD45 antibody (eBioscience) 10 min prior to euthanasia, as previously described (60). Lungs and nasal tissues were enzymatically digested for 1 h at 37 °C using Collagenase D (1 mg/mL; Sigma-Aldrich) and DNase I (20 U/mL; Sigma-Aldrich). Following red blood cell lysis, single-cell suspensions were incubated with CD16/CD32 FcγRIII (1:100) to block Fc receptors, stained with LIVE/DEAD Aqua (Invitrogen), and subsequently labeled with the following antibodies: CD45.2-BV650 (BD), CD3-PeCy7 (BD), CD4-FITC (Invitrogen), CD44-PE (BD), CD62L-PE-CF594 (BD), CD103-APC-EF780 (Invitrogen), and CD69-APC (Invitrogen). TRM CD4+ T cells were defined as CD45− CD44+ CD62L− CD69+ CD103+/− CD4+. Fluorescence minus one (FMO) and isotype control antibodies were used to validate gating. Flow cytometric acquisition was performed on an LSR Fortessa using Diva software (BD Biosciences), and data were analyzed with FlowJo (TreeStar). [file Image1.tif]
